# Silver Wire Amplifies the Signaling Mechanism for IL-1beta Production More Than Silver Submicroparticles in Human Monocytic THP-1 Cells

**DOI:** 10.1371/journal.pone.0112256

**Published:** 2014-11-14

**Authors:** Hye Jin Jung, Pyo June Pak, Sung Hyo Park, Jae Eun Ju, Joong-Su Kim, Hoi-Seon Lee, Namhyun Chung

**Affiliations:** 1 Department of Biosystems and Biotechnology, College of Life Sciences and Biotechnology, Korea University, Seoul, Korea; 2 Bioindustry Process Center, Jeonbuk Branch Institute, Korea Research Institute of Bioscience and Biotechnology, Jeoneup, Korea; 3 College of Agriculture and Life Science, Chonbuk National University, Jeonju, Korea; University of California, Merced, United States of America

## Abstract

Silver materials have been widely used in diverse fields. However, their toxicity and their mechanism, especially in different forms, have not been studied sufficiently. Thus, cytotoxicity, apoptosis, and interleukin-1beta (IL-1β) production were investigated using macrophage-like THP-1 cells in the presence of Ag microparticles (AgMPs, 2.7 µm), Ag submicroparticles (AgSMPs, 150 nm), and Ag wires (AgWs, 274 nm×5.3 µm). The levels of cytotoxicity, apoptosis, and IL-1β production by AgWs were higher than those by the other two AgSMPs and AgMPs. This trend was also observed with each step of the signaling mechanism for IL-1β production, which is a single pathway affiliated with ROS generation or lysosomal rupture or both, cathepsin B, caspase-1 (NALP3 inflammasome), and finally IL-1β production in THP-1 cells. All these results suggest that, for development of safe and effective silver materials, the shape or form of silver materials should be considered, especially for macrophage cell lines because epithelial cell lines are not overly sensitive to silver materials.

## Introduction

Advances in nanotechnology have promoted the use of merchandise containing silver materials with which the public can easily come into contact [1]. Indeed, silver materials are broadly made use of for industrial and biomedical applications since they possess remarkable antimicrobial activity [2], [3]. Therefore, the general public can easily be exposed to silver materials from diverse fields. Recently, some studies have demonstrated that silver materials introduced into the systemic blood supply can induce blood-brain barrier dysfunction and astrocyte swelling, in addition to causing neuronal degeneration [4]. Several studies have reported that silver materials significantly decrease mitochondrial function and induce cell necrosis or apoptosis of several cell types [3]. Furthermore, an *in vivo* study by Larese *et al*. has shown that silver materials can penetrate into the upper layers of the epidermis in excised human skin in static diffusion cells [5]. Another *in vivo* study reported that silver materials induce inflammatory responses and tissue damage in the lungs of mice.

Inflammation is a major biological response to harmful stimuli such as pathogens and irritants that occur during infections or after tissue damage [6]. IL-1β is related to some of cytokines, which cause a variety of biological effects associated with infection, inflammation, and autoimmune processes [7]. IL-1β is also an important proinflammatory mediator that is concerned with the generation of systemic and local responses to infection and injury [8]. There is evidence that IL-1β can induce apoptosis and cell proliferation in chondrocytes [9], that is, the inactive precursor pro-IL-1β in the cytosol is converted to mature IL-1β by caspase-1. Caspase-1 itself is synthesized as an inactive pro-caspase-1 (45 kDa zymogen) that undergoes autocatalytic processing in the presence of the stimuli. The activity of caspase-1 is tightly controlled by cytosolic multiprotein complexes called NALP3 inflammasomes (also called cryopyrin or NLRP3).

NALP3 inflammasomes are composed of Nod-like receptor protein NALP3, cardinal, adaptor ASC (apoptosis-associated speck-like protein containing a C-terminal caspase recruitment domain), and caspase-1. NALP3 inflammasomes potently modulate innate immune function by regulating the maturation and secretion of IL-1β [10], [11]. NALP3 inflammasome is assembled and activated in the presence of the pathogen-associated molecular patterns (PAMPs) and damage-associated molecular patterns (DAMPs). Though the mechanisms of NALP3 inflammasome activation remain unclear, two separate groups have recently reported on the mediator of NALP3 inflammasome activation. First, lysosomal destabilization and subsequent release of cathepsin B into the cytoplasm induced activation of NALP3 inflammasomes [12]. Second, phagocytosis of crystalline silica by macrophages led to reactive oxygen species (ROS) production, which induces activation of NALP3 inflammasomes [13].

Nowadays, AgWs, and not nanoparticles, are utilized by drug delivery systems [14], [15]. AgWs is more preferred for drug delivery system than other silver materials. Above all, AgWs have been suggested for use in nanoscale field-effect transistors, scanning probe microscopy tips, and sensing array elements [14]. In addition, AgWs have been applied in these fields, but even though AgWs are widely applied to living tissues, there is a serious lack of information on the signaling mechanism for the possible toxic effects of AgWs [16], [17]. Therefore, the present study aimed to elucidate the signaling mechanism for the cytotoxic effect of silver materials including AgWs, AgSMPs, and AgMPs. We found that the differential degrees of cytotoxicity as observed by apoptosis and IL-1β expression in human monocytic THP-1 cells are correlated to the observed signaling intensity. Our results might provide basic information that helps to design safe and effective forms of silver materials.

## Materials and Methods

### Cell culture

THP-1 cells (human acute monocytic leukemia cell line; TIB-202, ATCC, USA) were plated at a density of 3.0×10^5^ cells/mL with RPMI 1640 (WelGENE, Korea) supplemented with 10% heat-inactivated fetal bovine serum (FBS; JBI, Korea), 0.05 mM 2-mercaptoethanol, 100 U/mL penicillin, and 100 µg/mL streptomycin in 6-well culture plates. The culture was maintained in a 37°C, 5% CO_2_ atmosphere.

### Characterization of silver materials

The morphologies of silver materials were observed by field-emission scanning electron microscopy (FE-SEM; JEOL 7500, US), equipped with energy-dispersive X-ray spectroscopy (EDS). Each silver material was affixed to the mounts by carbon tape. We investigated the zeta potential of silver materials using the Nanoparticle size & zeta potential analyzer (90plus, Brookhaven Instruments, Germany). To measure the zeta potential, which are a surface electrical characteristic for probing the interaction between particles, all silver materials were freshly prepared in water, FBS, and serum-free RPMI 1640 to a concentration of 100 µg/mL [18].

### Treatment of silver materials

Silver materials used in this experiment were AgMPs (2.7 µm; Sigma-Aldrich, USA), AgSMPs (150 nm; Nano Technology Inc., Korea), and AgWs (274 nm×5.3 µm; NanoAmor, USA). THP-1 cells were seeded at a density of 1.0×10^6^ cells per well in a 6-well plate. The cells were differentiated into macrophage-like cells by adding 0.5 µM of phorbol 12-myristate 13-acetate (PMA; Sigma-Aldrich, USA) for 24 h prior to use. PMA-primed THP-1 cells were treated with AgWs, AgSMPs, or AgMPs. All silver materials were in serum-free RPMI 1640 to a concentration of 2 mg/mL prior to each experiment. The stock suspension was sonicated for 3 min to disperse silver materials, and then dilutions were made to achieve final test concentrations. The cells were treated with various concentrations (25, 50, 100, and 200 µg/mL) of silver materials according to the time schedule [19].

### Morphology of silver materials-treated cells

Cells treated with silver materials were photographed by light optical microscopy. After exposure to 100 µg/mL of silver materials for 24 h, THP-1 cells were gently washed with phosphate-buffered saline and then pictured under light optical microscopy (CK70, Olympus, Japan).

### WST-1 cytotoxicity assay

THP-1 cells (5.0×10^4^ cells/well) were differentiated by PMA for 24 h in a 96-well cell culture plate and were then washed with a cell culture medium. After differentiation into macrophage, cells were treated with silver materials for 24 h. The cytotoxicity assay was measured by the PreMix WST-1 Cell Proliferation Assay System (Takara Bio Inc., Japan) according to the manufacturer's protocol. Briefly, 10 µL of WST-1 reagent was added per well, and the cells were additionally incubated for 1 h. The absorbance of WST-1-added samples was measured by an EL800 microplate reader (Bio-Tek Instruments, USA) at 490 nm.

### Lactate dehydrogenase (LDH) release assay

PMA-primed THP-1 cells were treated with silver materials and evaluated for cytotoxicity using an LDH Cytotoxicity Detection Kit (Takara Bio Inc., Japan) according to the manufacturer's protocol. Supernatant of silver materials-treated cells was separated by centrifugation and analyzed for LDH release. Samples were measured using a microplate reader at a wavelength of 490 nm.

### Analysis of sub-G1 DNA content and cell size

DNA fragmentation was analyzed by flow cytometer at an excitation wavelength (Ex) of 488 nm and emission wavelength (Em) of 610 nm. Briefly, after 24 h of exposure to silver materials, the PMA-primed THP-1 cells were collected. Then, the cell pellet was washed in PBS, fixed in ice-cold ethanol (70%), and stored at −20°C for 2 h or longer. Before flow cytometry analysis, ethanol was removed by centrifugation and the cells were washed twice with PBS. The cell pellet was resuspended in a minimal amount of PBS and stained with propidium iodide (PI, 50 µg/mL; Sigma-Aldrich) staining solution containing RNase (0.1 mg/mL; Sigma-Aldrich) and Triton X-100 (0.1%, v/v; Sigma-Aldrich). The PI-stained cells were incubated at 37°C in the dark for 30 min. The sub-G1 DNA content was obtained using the flow cytometer by measuring the amount of PI-labeled DNA in fixed cells. Data was analyzed using the ModiFit LT software program (Verity Software House, USA).

### Flow cytometer analysis for apoptosis

PMA-primed THP-1 cells were treated with various concentrations of silver materials for 6 h. After treatment, cells were detached and harvested by centrifugation. Cells were then resuspended in an Annexin V binding buffer, a component of the Apoptosis Detection kit (BD Biosciences, USA). Cells were then mixed with 5 µL of Annexin V-FITC and 5 µL of PI solution. The cell suspension was incubated in the dark for 15 min at room temperature. The fluorescence intensity of Annexin V-FITC and PI was analyzed at an Ex/Em of 488/530 nm and 488/617 nm, respectively.

### Enzyme-linked immunosorbent assay (ELISA)

PMA-primed THP-1 cells were treated with various concentrations of silver materials for 24 h. IL-1β production levels in the culture medium were measured using an ELISA kit (R&D Systems, USA) according to the manufacturer's protocol. Plates were read at 450 nm, using an EL800 microplate reader. CA-074-methyl ester (CA-074-Me), diphenyleneiodonium chloride (DPI), and butylated hydroxyanisole (BHA) were purchased from Sigma-Aldrich. For analysis of inhibitor effects, PMA-primed THP-1 cells were washed with RPMI 1640 and pre-incubated with CA-074-Me, DPI, and BHA for 30 min. Then, the cells were treated with each silver material for 24 h.

### Reverse transcription polymerase chain reaction (RT-PCR)

PMA-primed THP-1 cells (1×10^7^) were treated with 100 µg/mL of silver materials for 24 h and then, all RNA was extracted from the cells using an RNeasy Mini Kit (Qiagen, Germany) in accordance with the manufacturer's protocol. Extracted RNA was reverse transcribed using the Reverse Transcription System (Promega, USA). Synthesized cDNA was amplified by PCR using *Taq* polymerase (GeneAll, Korea). The sequence of specific primers for NALP3 (cytoplasmic receptor) and GAPDH was as follows: NALP3 forward: 5′-TGCCTTTGACGAGCACATAG-3′; NALP3 reverse: 5′-GCAGCAAACTGG AAGGAAG-3′; caspase-1 forward: 5′-GAAGGCATTTGTGGGAAGAA-3′; caspase-1 reverse: 5′-CATCTGGCTGCTCAAATGAA-3′; and GAPDH forward: 5′-GAGTCAACGGATTTGGTCGT-3′; GAPDH reverse: 5′-TTGATTTTGGAGGGATCTCG-3′. After 5 min at 95°C, 28 cycles at 95°C for 1 min, 53°C for 1 min (caspase-1; 51°C), and 72°C for 2 min were performed, ending with a final extension step at 72°C for 5 min. Quantification of PCR products was performed by electrophoresis. Data was analyzed using the Image Quant software program (GE Healthcare, USA).

### Measurement of caspase-1 enzymatic activity

Caspase-1 enzymatic activity was determined by a caspase-1 colorimetric assay kit (R&D Systems, US) according to the manufacturer's protocol. In brief, the PMA-primed THP-1 cells (1×10^7^) were treated with 200 µg/mL of silver materials for 24 h, and then the cells were lysed in 250 µL of cold lysis buffer. The cell lysates were incubated on ice for 10 min and centrifuged at 10,000×g for 1 min, and then the supernatants were collected. A volume of 50 µL of cell lysate was added to 50 µL of caspase-1 reaction buffer with 40 mM dithiothreitol (DTT). Each sample was combined with 5 µL of 4 mM caspase-1 colorimetric substrate (WEHE-pNA), followed by a 2-h incubation at 37°C. The enzymatic activity of caspase-1 was measured by an EL800 microplate reader at 405 nm [20].

### Acridine orange staining

Change in lysosomal permeability was measured by acridine orange (Sigma-Aldrich) staining using flow cytometry [21]. Briefly, PMA-primed THP-1 cells were preloaded with 0.5 µg/mL acridine orange in RPMI 1640 for 30 min at 37°C and then washed three times with RPMI 1640 and treated with silver materials for 24 h. After exposure, cells were detached and harvested. The fluorescence intensity was analyzed using a FACSCalibur flow cytometer at an Ex/Em of 488/620 nm. Fluorescent photomicrographs of lysosomes in THP-1 cells upon exposure to silver materials were examined using a fluorescence microscope (Axio Observer D1, Carl-Zeiss, Germany). The cells were washed 3 times with phosphate buffer saline after incubation with silver materials and were stained with 20 µg/mL acridine orange for 15 min.

### Measurement of cellular ROS production

Intracellular formation of ROS was measured as described previously using oxidation-sensitive dye 2′,7′-dichlorofluorescin diacetate (DCFH-DA) as the substrate [22]. THP-1 cells growing in black 96-well microplates were loaded with 100 µM DCFH-DA in phosphate-buffered saline and incubated for 30 min in the dark. Cells were then treated with different concentrations of each silver material and incubated for 24 h after washing the cells with phosphate-buffered saline three times. The formation of 2′,7′-dichlorofluorescin (DCF) due to oxidation of DCFH in the presence of various ROS was read every 30 min at an Ex of 485 nm and an Em of 535 nm using a multilabel plate reader (Victor3, Perkin Elmer, USA). Fluorescent photomicrographs of ROS in THP-1 cells upon exposure to silver materials were obtained using a fluorescence microscope. The cells were washed 3 times with PBS after incubation with silver materials and were stained with 40 µg/mL DCFH-DA for 30 min.

### Statistical analyses

Data were expressed as mean ±SD. The data were analyzed using Student's t-test where statistical significance was calculated for silver treated samples against untreated (control). Statistical significance was determined in level of *p<0.05 or **p<0.01.

## Results

### Particle characterizations

**Figure 1:**
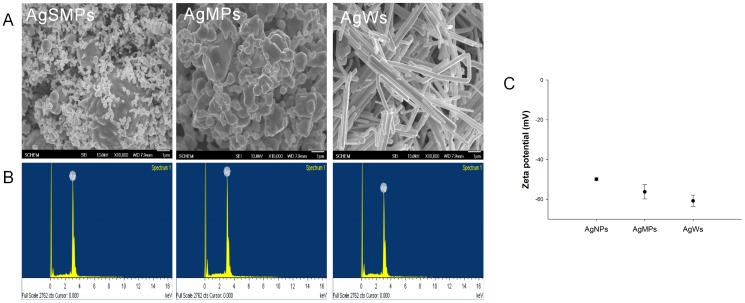
Characterization of silver materials. (A) FE-SEM top-view images of silver materials (10,000×). (B) EDS. (C) Zeta potential of silver materials in serum-free RPMI 1640.

The shapes of silver materials were examined using FE-SEM images (Fig. 1A). The images showed that AgMPs are larger than AgSMPs and that AgWs have an expected aspect ratio of about 19. Purity of the particles was confirmed by ion analysis of silver materials using EDS. The major constituent of silver materials was silver (Fig. 1B) and the atomic percentages of the particles were 100% (data not shown). We investigated the zeta potential to confirm the stability of silver materials in water, FBS, serum-free RPMI 1640. If particles have high zeta potentials of the same polarity, this can prevent agglomeration among particles because of the repulsive force between each particle. Values for zeta potential were similar in three solvents. Therefore, stability of silver materials was estimated in serum-free RPMI 1640 that was used for this present experiment. The values of silver materials were less than −40 mV (AgWs: −60.82±2.89 mV; AgSMPs: −49.91±0.76 mV; AgMPs: −56.26±3.60 mV) (Fig. 1C). Therefore, the particles were stable in the serum-free RPMI 1640.

### Induction of cytotoxicity by silver materials

**Figure 2:**
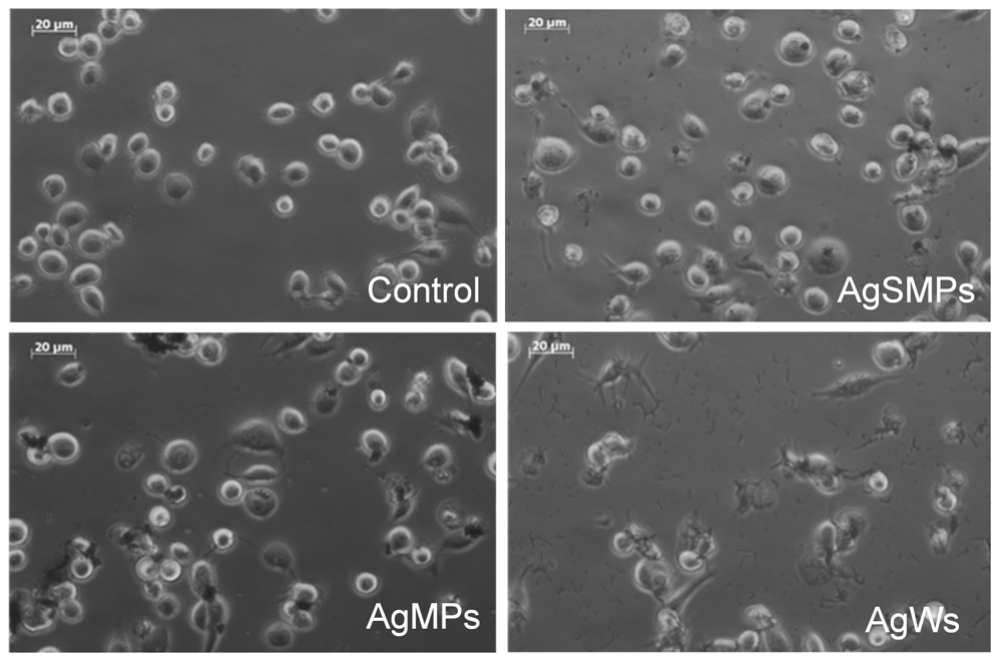
Phase-contrast micrograph of THP-1 cells morphology in both the absence and presence of silver materials. PMA-primed THP-1 cells were treated with 100 µg/mL of silver materials for 24 h. The cells were photographed using a phase-contrast microscope to estimate overall morphology (400×).

To assess the cytotoxicity of silver materials on THP-1 cells, overall morphologies of PMA-primed THP-1 cells in both the absence and presence of different forms of silver materials were observed using a phase-contrast microscope (Fig. 2). Unlike control, the cells cultured with AgWs were more elongated or had more of a crushed morphology than those cultured with the other silver materials.

**Figure 3:**
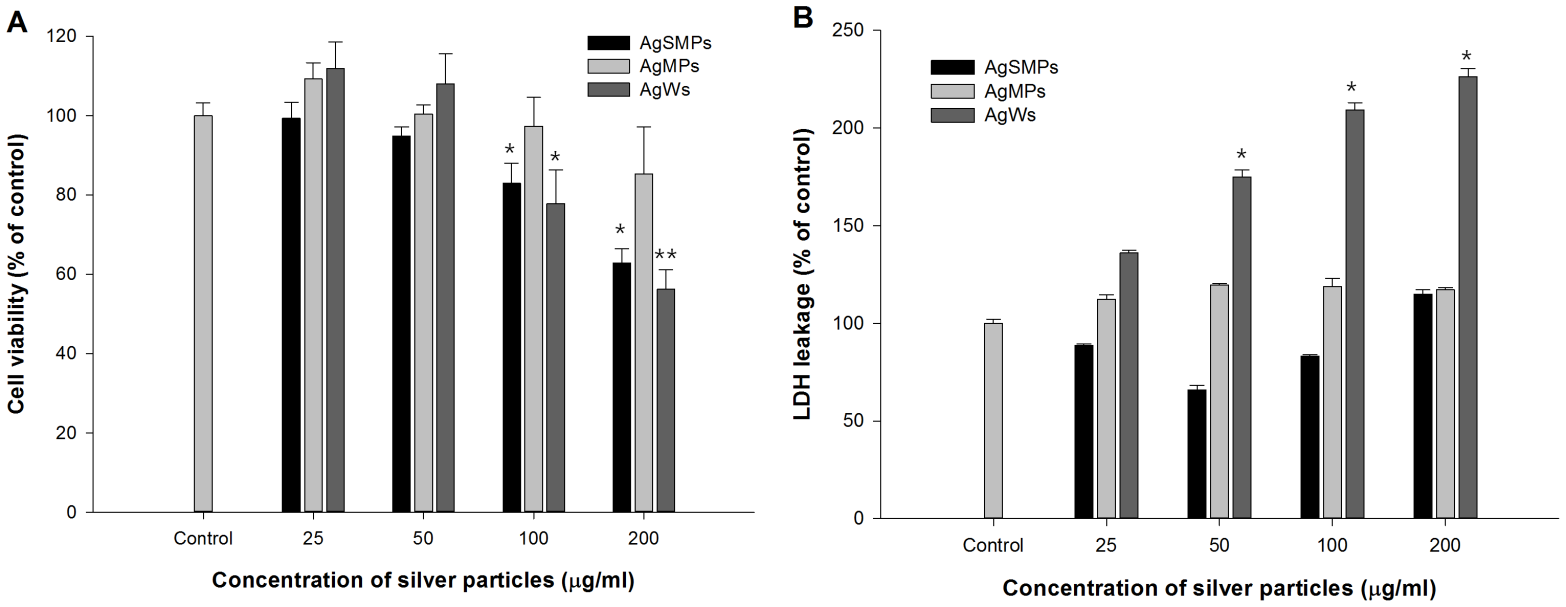
Cytotoxicity induced by different forms of silver materials. (A) Cell viability was measured by WST-1 assay. PMA-primed THP-1 cells were treated with different forms of silver materials for 24 h and the extents of viability for the treated groups (25, 50, 100, and 200 µg/mL) were expressed as a percentage of the control group. Data are represented as the mean ±SD (n = 5). (B) LDH leakage assay due to membrane damage was measured using an LDH Cytotoxicity Detection Kit. PMA-primed THP-1 cells were treated with different forms of silver materials for 24 h and the extents of LDH leakage for the treated groups were expressed as a percentage of the control group. Data are represented as the mean ±SD (n = 5). *p<0.05, **p<0.01.

Cell proliferation in the presence of silver materials was measured by WST-1 assay (Fig. 3A). As the concentration of silver materials increased (25–200 µg/mL) for 24 h, the viability of PMA-primed THP-1 cells decreased gradually. The degree of cytotoxicity in higher concentrations (100 and 200 µg/mL) decreased more with AgWs and AgSMPs than with AgMPs. Next, extracellular LDH levels were measured to evaluate the cell membrane damage elicited by silver materials (Fig. 3B). As the concentration of silver materials increased, the LDH level of PMA-primed THP-1 cells became much higher with AgWs than with the others. This result suggests that AgWs induced more membrane damage or LDH leakage than the others.

### Induction of apoptosis by silver materials

**Figure 4:**
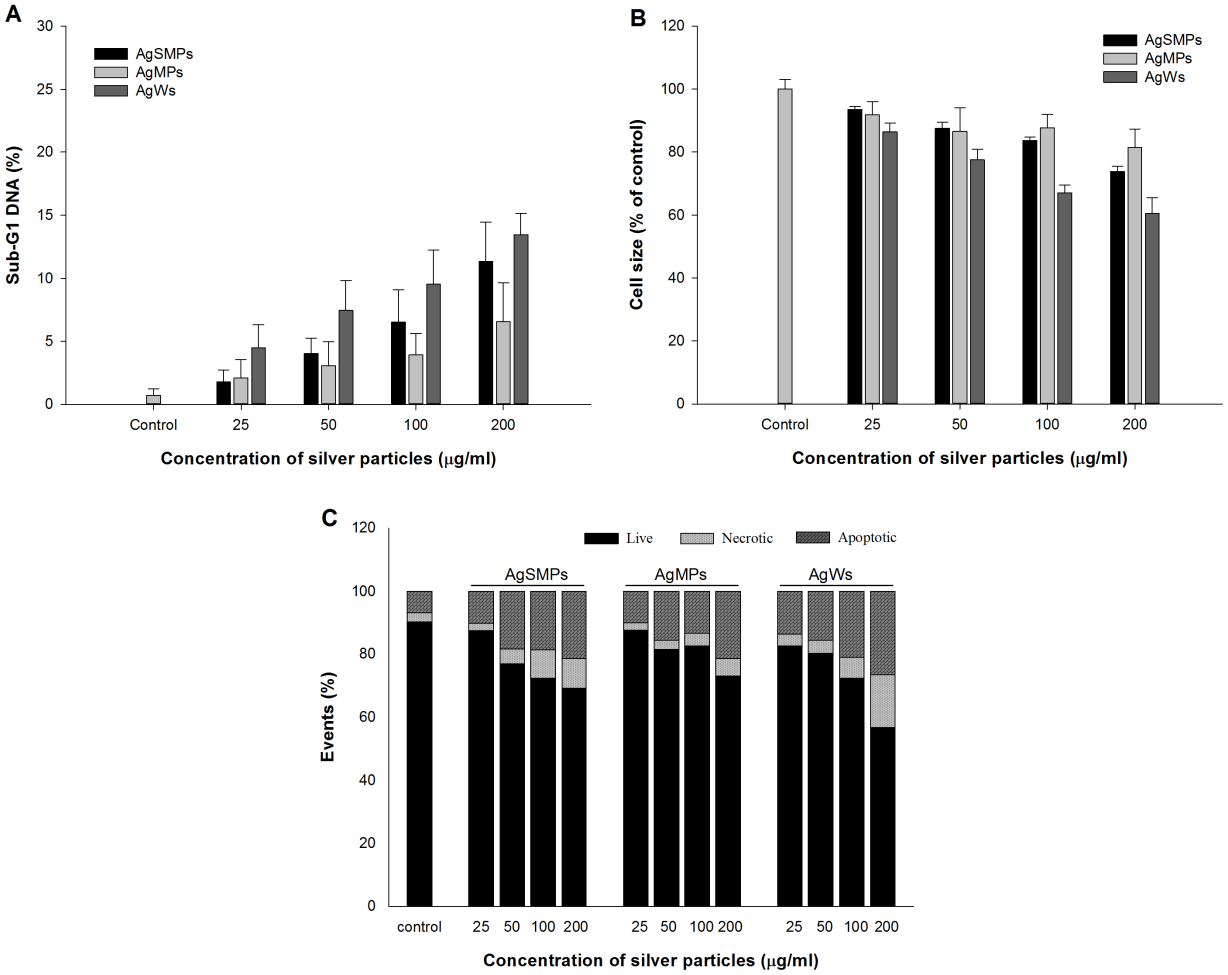
Various evidences of cell death induced by different forms of silver materials. (A) The Sub-G1 DNA analysis for PMA-primed THP-1 cells after treatment with silver materials for 24 h. The extent of the sub-G1 peak was determined by flow cytometry using PI staining. Data are represented as the mean ±SD (n = 3). (B) The decrease in the extent of relative cell size as measured using forward scattering of flow cytometry. PMA-primed THP-1 cells were treated with different forms of silver materials for 24 h. Data are represented as the mean ±SD (n = 3). (C) Annexin-V staining of PMA-primed THP-1 cells to detect the mode of cell death in the presence of different forms of silver materials for 6 h. AnnexinV/PI analysis was performed to assess the percentage of viable, apoptotic, and necrotic cells.

Cell death induced by silver materials could occur either by an abrupt process called necrosis or by a tightly regulated process called apoptosis [23]. Characteristics of cell death by silver materials can be detailed by studying DNA damage, cell shrinkage, and apoptosis. DNA damage was detected by measuring sub-G1 DNA in cell cycle analysis. The results revealed that the extents of sub-G1 DNA were increased in a dose-dependent manner (Fig. 4A). The extents of sub-G1 DNA with different forms of silver materials were in the overall order of AgWs>AgSMPs>AgMPs. A significant decrease in the extent of forward scatter (indication of cell shrinkage) in flow cytometry was monitored after treatment with different types of silver materials. A dose course study showed that the treatment caused a rapid decrease in cell size, the degree of which was in the overall order of AgWs>AgSMPs≈AgMPs (Fig. 4B). Cells with irreversible damage undergo apoptosis. Apoptosis has been regarded as a major mechanism for cell death upon exposure to silver materials [24]. To unveil the extent and mode of cell death, annexin-V/PI staining and FACS analysis were performed (Fig. 4C). The results showed that the extent of apoptotic and necrotic cells increased in a dose-dependent manner with all types of silver materials. However, the rates of cells undergoing apoptosis were obviously higher than the rates of cells undergoing necrosis.

### Induction of IL-1β maturation and caspase-1 activation by NALP3 inflammasome in the presence of silver materials

**Figure 5:**
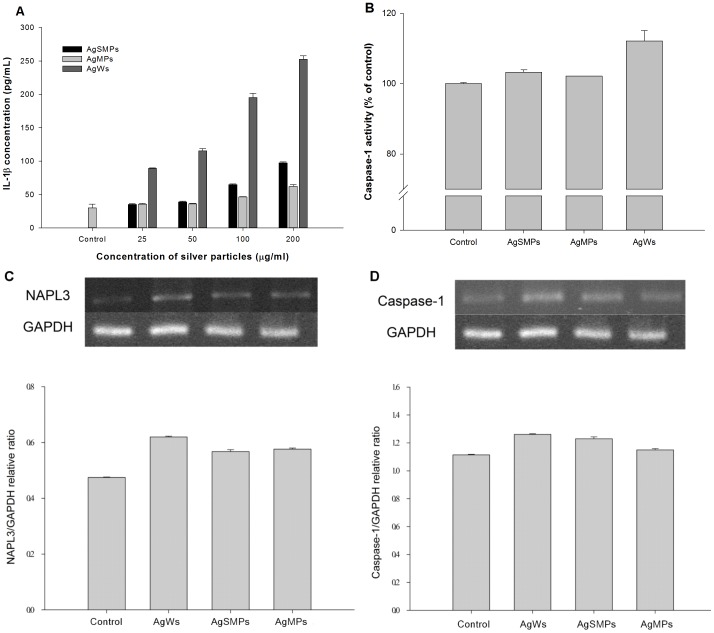
Extent of IL-1β production and active caspase-1 by PMA-primed THP-1 cells in the presence of silver materials. (A) Differences in the levels of IL-1β production induced by different forms of silver materials. The cells were treated with different silver materials for 24 h. Data are represented as the mean ±SD (n = 3). (B) Difference in the levels of caspase-1 activity. The cells were treated with 200 µg/mL of silver materials for 24 h. Data are represented as the mean ±SD (n = 3). (C) Differences in the levels of caspase-1 mRNA expression levels. The cells were treated with 100 µg/mL of silver materials for 24 h. (D) Differences in the levels of caspase-1 mRNA expression. The cells were treated with 100 µg/mL of silver materials for 24 h.

Proinflammatory response including release of mature IL-1β was induced by inhalation of AgSMPs [25]. Additionally, IL-1β production depended dramatically on the characteristics of silver materials [26]. Based on these two pieces of information, the association between the characteristics of silver materials and inflammatory response was hypothesized. THP-1 cells were incubated using different silver materials and the level of IL-1β production was analyzed with ELISA. PMA-primed THP-1 cells responded dose-dependently to the addition of silver materials, with AgWs inducing much higher IL-1β production than the other two particles at all concentrations tested (Fig. 5A).

The protease caspase-1, which regulates the cleavage and maturation of the precursor form of IL-1β, is made up of NALP3 inflammasome complex [8], [27]. After assembly of NALP3 inflammasome, caspase-1 regulates IL-1β maturation. On the other hand, NALP3 inflammasome is required for the activation of procaspase-1 in response to several types of danger signals. We hypothesized that the extent of IL-1β induction is affected by the differential activity of caspase-1 in the presence of silver materials. Caspase-1 enzyme activity was measured in the cell lysate. As a result, although the difference in the extent was small, caspase-1 activity was higher with AgWs than with the others (Fig. 5B). We also examined the mRNA gene expression extent of caspase-1 and NALP3 by PMA-primed THP-1 cells in the presence of silver materials. We found that caspase-1 gene expression was higher with AgWs than with the others (Fig. 5C). Furthermore, NALP3 mRNA gene expression was higher with AgWs than with the others (Fig. 5D). These findings indicate that the level of NALP3 gene expression has relevance to the level of caspase-1 enzyme activation and gene expression. These results collectively indicate that AgWs activates the NALP3 inflammasome complex more than the others, thus leading to the stronger activation of caspase-1.

### Activation of NALP3 inflammasome via lysosomal destabilization and cathepsin B activation in the presence of silver materials

**Figure 6:**
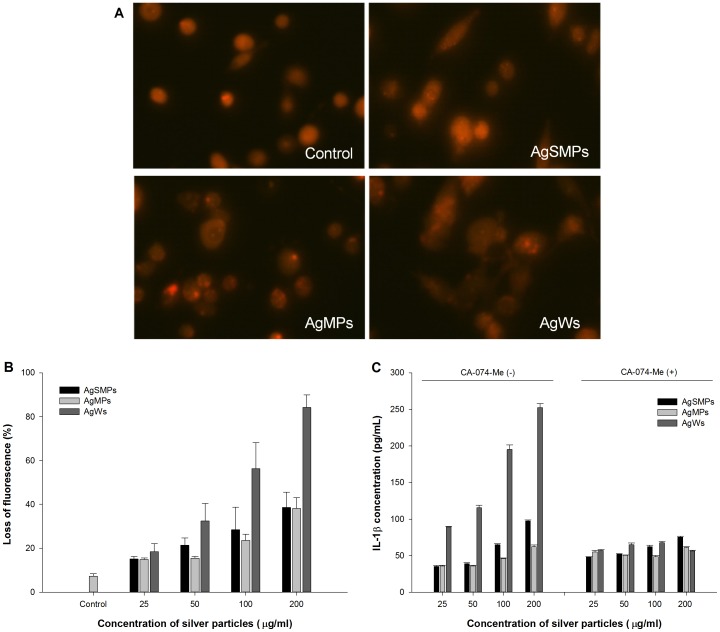
Involvement of lysosomal destabilization and cathepsin B activity with IL-1β production in the presence of silver materials. (A) PMA-primed THP-1 cells were treated with each of the silver materials (100 µg/mL) for 24 h. At the end of exposure, the cells were loaded with acridine orange (20 µg/mL) for 15 min at 37°C and photographed using a fluorescence microscope (400×). (B) Lysosomal destabilization as measured by loss of fluorescence with increasing concentration of silver materials. The cells were preloaded with acridine orange (0.5 µg/mL) for 30 min, treated with silver materials, and analyzed using FACS to determine the loss of acridine orange from lysosome. Data are represented as the mean ±SD (n = 3). (C) Involvement of cathepsin B in silver materials-induced IL-1β production. The cells were treated with different forms of silver materials for 24 h in both the absence and presence of cathepsin B inhibitor (CA-074-Me; 10 µM). IL-1β levels were measured using ELISA. Data are represented as the mean ±SD (n = 3).

The activator of NALP3 inflammasome, for example crystalline or particulate materials, causes phagocytosis and then leads to lysosomal damage, resulting in cytosolic release of lysosomal contents [8]. Cathepsin B, a lysosomal protease, triggers the NALP3 inflammasome directly. To evaluate the lysosomal destabilization by silver materials, cell staining was conducted with acridine orange and pictured using a fluorescence microscope (Fig. 6A). Acridine orange reacts with the acidic content of organelles after the silver materials incurs membrane damage and then the fluorescence intensity decreases. As can be observed in Fig. 6A, the fluorescent intensity with AgWs was lower or weaker than that with the others. The fluorescent intensity of PMA-primed THP-1 cells treated with silver materials showed a very similar trend (Fig. 6B). These results demonstrate that different forms of silver materials induced the destabilization of lysosomal membranes dose-dependently and that AgWs caused more lysosomal membrane damage than the others.

Next, we hypothesized that cathepsin B was involved in the process of activation of NALP3 inflammasome and caspase-1. PMA-primed THP-1 cells were treated with silver materials in both the absence and presence of a cathepsin-B-specific inhibitor (CA-074-Me). The presence of CA-074-Me significantly suppressed the extent of IL-1β production in all concentrations tested, regardless of the type of silver materials (Fig. 6C). However, in the absence of CA-074-Me, the extent of IL-1β production increased in a dose-dependent manner and was in the order of AgWs>AgSMPs>AgMPs. This result suggests that more cathepsin B was released into the cytoplasm with AgWs than with the others, resulting in the higher activation of NALP3 inflammasome and IL-1β production with AgWs than with the others.

**Figure 7:**
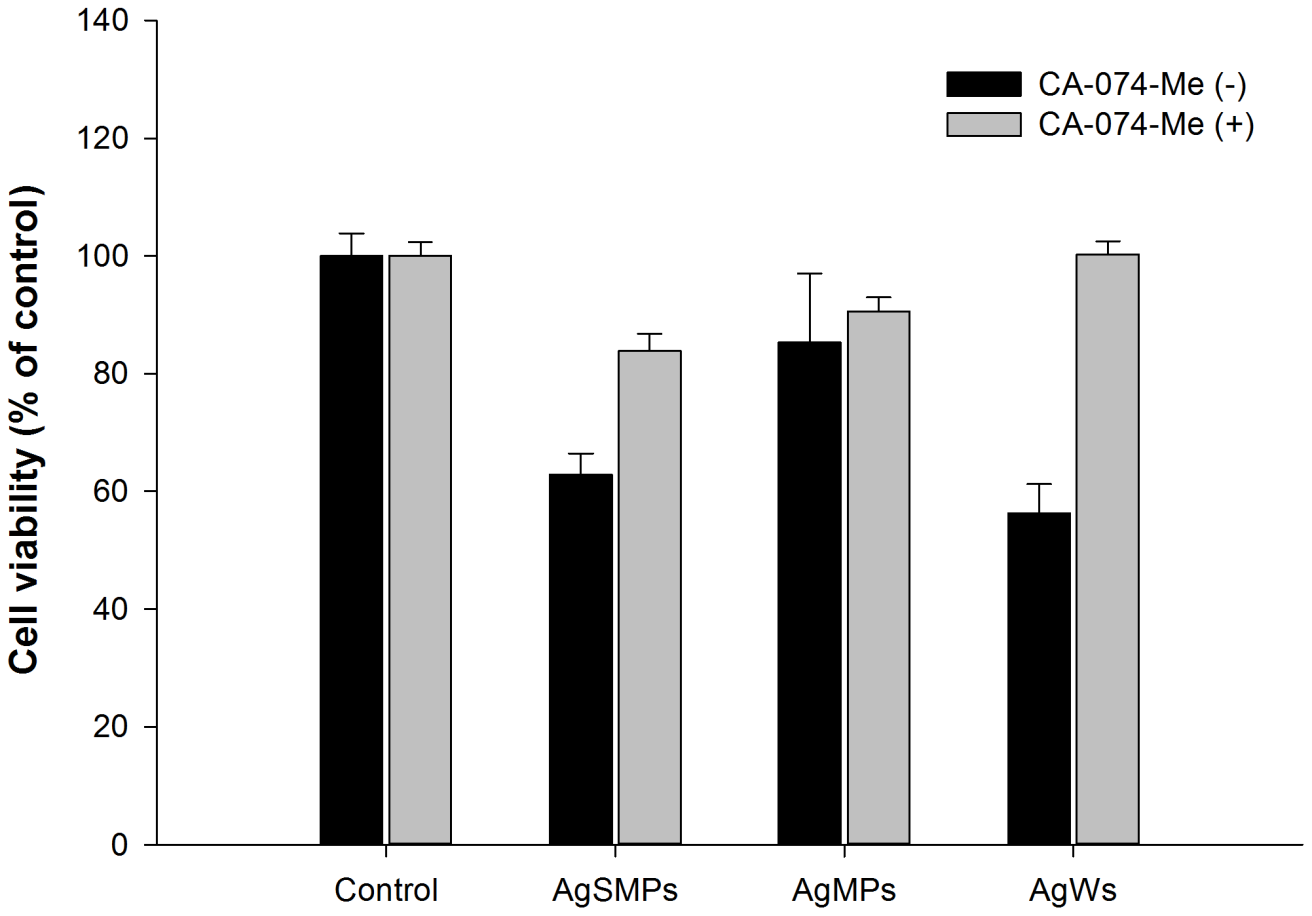
Involvement of cathepsin B in cytotoxicity induced by silver materials. Cell viability was measured by WST-1 assay. PMA-primed THP-1 cells were treated with 200 µg/mL of each of the silver materials for 24 h in both the absence and presence of cathepsin B inhibitor (CA-074-Me; 10 µM). Data are represented as the mean ±SD (n = 5). *p<0.05.

Additionally, PMA-primed THP-1 cells were treated with silver materials both with and without CA-074-Me. Then, WST-1 assay was performed to observe cell viability (Fig. 7). The results demonstrate that CA-074-Me significantly suppressed the cytotoxicity by silver materials, especially in the presence of AgWs. These data suggest that cathepsin B released from damaged lysosome by the action of AgSMPs or AgWs was a cause of the observed phenomenon.

### Correlation between ROS generation and NALP3 inflammasome activation

**Figure 8:**
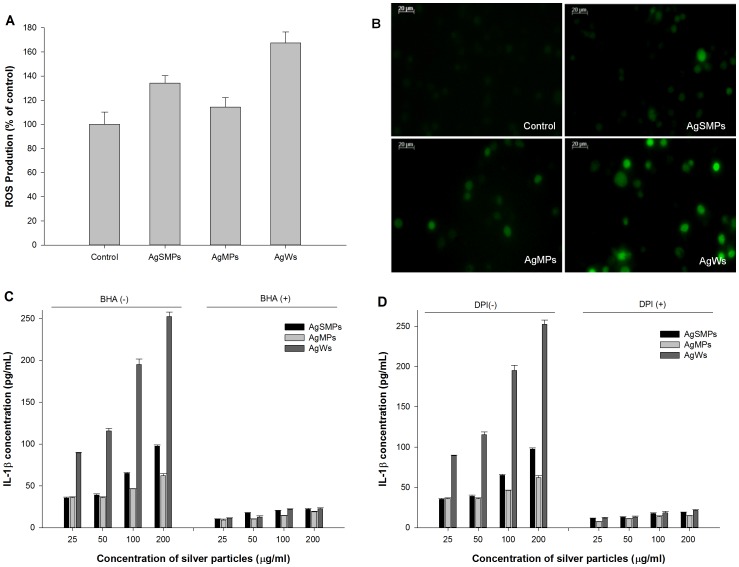
The association between silver materials-induced ROS generation and IL-1β production. (A) PMA-primed THP-1 cells were treated with each of the silver materials (100 µg/mL) for 24 h. At the end of exposure, the cells were loaded with DCFH-DA (100 µM) for 30 min and the fluorescence intensity was measured using a fluorometer. Data are represented as the mean ±SD (n = 5). (B) The cells were treated with each of the silver materials (100 µg/mL) for 24 h. At the end of exposure, the cells were loaded with DCFH-DA (40 µM) for 30 min at 37°C and photographed using a fluorescence microscope (400×). (C) The cells were treated with different forms of silver materials for 24 h in both the absence and presence of BHA (150 µM). IL-1β levels were measured using ELISA. Data are represented as the mean ±SD (n = 3). (D) The cells were treated with different forms of silver materials for 24 h in both the absence and presence of DPI (200 µM). IL-1β levels were measured using ELISA. Data are represented as the mean ±SD (n = 3).

Like cathepsin B, ROS are widely linked to signaling pathways and are known to induce activation of NALP3 inflammasome. To test the hypothesis that the generation of ROS correlates with toxicity and inflammatory response by different types of silver materials, the relationship between the level of ROS generation and the IL-1β production was investigated in the both the absence and presence of ROS inhibitors. First, the levels of ROS with PMA-primed THP-1 cells were measured in the presence of different forms of silver materials (Fig. 8A). The results showed that, in the presence of silver materials, more ROS was produced than with control and that the extent of ROS production was in the order of AgWs>AgSMPs>AgMPs. Photographs taken using a fluorescence microscope (Fig. 8B) supported the previous data in Fig. 8A.

ROS inhibitor BHA (a broad ROS scavenger) or DPI (a specific inhibitor of NADPH oxidase) was employed to suppress ROS production by PMA-primed THP-1 cells in the presence of silver materials. Then, when IL-1β production was measured, IL-1β production was suppressed significantly more in the presence of ROS inhibitors than in the absence of ROS inhibitors (Figs. 8C and D). The extent of IL-1β production was in the order of AgWs>AgSMPs>AgMPs. Considering the report that found that NADPH oxidase generated inflammasome-activating ROS [10], these results imply that ROS as well as cathepsin B are linked to the signaling pathway of IL-1β production through NALP3 inflammasome in the presence of silver materials, especially AgWs or AgSMPs.

## Discussion

Engineered silver materials, such as AgSMPs and AgWs, are being developed and used in a variety of applications [28], and thus, people can easily be exposed to these silver materials. However, what is still required is a) a detailed description of the molecular mechanism induced by silver materials and b) a risk assessment for using them. This present study aimed to identify the mechanisms of inflammatory responses to silver materials, including AgWs. Many previous studies reported about the risks of wire-structured metal. AgWs induced cytotoxicity at concentrations ≥190 µg/mL in Hela cells and HEp-2 cells and resulted in the up-regulation in macrophages of inflammatory cytokines such as IL-1α and IFN-γ and the down-regulation of IL-6 [29], [30]. Also PVP-coated Ag wire causes cytotoxicity but that PVP-coated spherical Ag has no cytotoxicity in A549 cell lines [31].

Based on these previous studies, we employed AgSMPs of 150 nm in the present study. As expected and known in other studies, if the particle size become smaller than 150 nm, we believe that the cytotoxicity of sphere-shaped particle increases up to certain point since many studies indicated that nanoparticle with too small size (for example, <10 nm) is not toxic any more [32]. So, we hypothesized that AgWs might induce more cytotoxicity and inflammatory responses than AgSMPs and AgMPs.

In the present study, silver materials were diluted and sonicated before addition to the culture medium RPMI 1640 containing 10% FBS. It is known that silver materials can be stabilized in the presence of FBS [33]. Thus, complexation between silver materials and FBS may help silver materials to be stabilized and dispersed so that silver materials can enter inside the macrophage cells. In analogy, silver materials in the environment are not supposed to be coated. However, when the silver materials come into contact with human cells, they are exposed to human serum to be coated. On the other hand, silver materials containing no surface modifiers or stabilizers could enter the macrophage cells by pinocytosis [3]. In a cytotoxicity test of selected silver materials, the cytotoxicity measured by WST-1 and LDH release assay noticeably decreased and increased, respectively, in a dose-dependent manner. In particular, the cells treated with AgWs exhibited the highest degree of cytotoxicity and membrane damage (Fig. 3). As shown in Fig. 4A, all types of silver materials elicited DNA fragmentation as evidenced by occurrence of a sub-G1 peak, and again AgWs induced a higher degree of sub-G1 DNA content than the others. Cell shrinkage, a hallmark of apoptotic cell death, showed almost the same trend after exposure to silver materials (Fig. 4B). As apoptosis has been suggested as a major mechanism for cell death by silver materials [34], we attempted to determine the mode of cell death (Fig. 4C). The data indicated that although apoptosis is the major mode of cell death, necrosis might be the second mode of cell death in the presence of silver materials, especially in the presence of a high concentration of AgWs.

IL-1β production via caspase-1 is presently considered to play a crucial role in initial inflammation [9], [35]. Both IL-1β production and caspase-1 activation were the highest with AgWs among the tested silver materials (Figs. 5A, B, and C). Caspase-1, a component NALP3 inflammasome, induces release of active IL-1β only after NALP3 inflammasomes are activated by environmental irritants [36]. As expected, NALP3 mRNA expression levels were highest with AgWs among the silver materials tested (Fig. 5D). The mechanism for NALP3 inflammasome-mediated IL-1β secretion by silver materials is not yet known, making the creation of safe silver materials difficult. A few studies reported that cathepsin B leakage after lysosomal rupture and cytoplasmic ROS play a critical role in the activation of NALP3 inflammasome [10], [12]. Thus, we demonstrated here that silver material-induced IL-1β production was mediated by cathepsin B release and ROS production (Figs. 6, 7, and 8). AgWs caused more serious damage to lysosomal membrane than the others (Figs. 6A and 6B). The specific inhibitor of cathepsin B, CA-074-Me, suppressed IL-1β production by THP-1 cells more evidently in the presence of AgWs than in the presence of the others (Fig. 6C), indicating that active cathepsin B is one of the most important activators of the inflammasome upon stimulation by silver materials.

The NALP3 activators, including ATP, asbestos, and silica, trigger the generation of ROS. Treatment with various ROS scavengers blocks NALP3 activation [11]. In the present study, AgWs generated more intracellular ROS than the others (Figs. 8A and B). Figs. 8C and D proved that silver material-induced IL-1β production is related to this increase in intracellular ROS. This result indicated that not only cathepsin B released from lysosome but also ROS generated by silver materials play crucial roles and possibly interact with each other in silver materials-induced IL-1β production. We believe that the silver materials are phagocytosized. Then, while producing ROS, the reactive silver material surface may interact with phagolysosomal membranes, leading to lysosomal rupture. However, as evidenced with the higher LDH values, AgWs might cause more membrane damage and rupture and more ROS production than AgSMPs (150 nm; nominal surface area  = 7.065×10^−2^ µm^2^) and AgMPs (2.7 µm; nominal surface area  = 2.289×10^−2^ µm^2^) since AgWs (274 nm×5.3 µm; nominal surface area  = 4.677 µm^2^) have more surface area than the others. As further study, we plan to elucidate on how cytotoxicity and signaling intensity are affected by the aspect ratio between the diameter and length of AgWs.

## Conclusions

In summary, we suggest that silver materials induce different levels of IL-1β production and cytotoxicity depending on their form. Furthermore, AgWs induced more apoptosis and IL-1β production than AgSMPs and AgMPs because AgWs cause more membrane damage than the others. We also speculated that silver material-induced IL-1β production is affected by a single pathway affiliated with ROS and lysosomal rupture, cathepsin B, caspase-1 (NALP3 inflammasome), and IL-1β production in THP-1 cells. For our healthy lives, the development of safe and effective metal materials should be advanced. However, it is necessary to obtain more information about the association between the shape of metal materials and their biological effects. These results may provide essential basic information for the development of safe forms of silver materials.
